# Airway Ultrasound: Applications in Pediatric Anesthesiology and Critical Care

**DOI:** 10.14740/jocmr6635

**Published:** 2026-08-26

**Authors:** Nahida Akhter, Tariq Wani, Joseph D. Tobias

**Affiliations:** a Department of Anesthesiology & Pain Medicine, Nationwide Children’s Hospital, Columbus, OH, USA; b Department of Anesthesiology & Pain Medicine, The Ohio State University College of Medicine, Columbus, OH, USA

**Keywords:** Point-of-care ultrasound, Airway management, Endotracheal intubation, Airway assessment, Cricothyrotomy

## Abstract

The clinical uses and potential applications of point-of-care ultrasound (POCUS) have seen a significant increase in the practice of anesthesiology and critical care medicine. While initially used for regional anesthesia and vascular access, POCUS has since expanded to have significant applications for airway management and evaluation. Ultrasound has been effectively used to visualize the anatomical structures of the hypopharynx and anterior neck including the cricothyroid membrane, tracheal rings, esophagus, and aerated lung in a simple, rapid, and non-invasive manner. POCUS has demonstrated utility across multiple aspects of airway management including preoperative airway assessment, identification and evaluation of the difficult airway, confirmation of endotracheal intubation, verification of cuff position in relation to the cricoid ring, endotracheal tube depth, and localization of the cricothyroid membrane for emergency airway access. This educational review outlines the novel uses of POCUS in airway evaluation and management in various clinical scenarios with a focus on the pediatric-aged patient.

## Introduction

Over the past decade, point-of-care ultrasound (POCUS) has seen a significant increase in utilization within anesthesiology and critical care medicine. Training in POCUS is now included in most curricula for trainees across both specialty tracks. While initially utilized primarily for central venous access and regional anesthesia, POCUS has expanded into various diagnostic and therapeutic domains. Current clinical applications include facilitation of peripheral venous access, assessment of gastric volume and aspiration risk, regional anesthesia (peripheral and neuraxial blockade), confirmation of one-lung ventilation (OLV), identification of pneumothorax or pleural effusions, and detection of intra-abdominal bleeding during trauma care [1–3]. More recently, ultrasound has been increasingly studied as a modality for airway evaluation and management across various clinical scenarios [4, 5]. It enables identification of airway anatomy including the bones and soft tissues of the hypopharynx and anterior neck, the cricothyroid membrane (CTM), tracheal cartilages, esophagus, and aerated lung in a simple, rapid, and non-invasive manner.

The applicability and advantages of POCUS have been demonstrated in various aspects of airway management, and these applications continue to expand (Table 1). This educational review outlines the novel use of POCUS in airway evaluation and management with a focus on pediatric-aged patients. The literature regarding the applications of POCUS across various clinical scenarios is reviewed including assessment of the airway and identifying the potentially difficult or compromised airway, confirmation of endotracheal intubation, documentation of appropriate endotracheal tube (ETT) placement including depth of placement, identification of the correct site for potential cricothyroidotomy, and documentation of effective lung isolation during OLV.

**Table 1:** Ultrasound for Airway Evaluation and Management

1. Assessment of the airway
a. Airway assessment and identifying the difficult airway
b. Grading or identification of obstructive sleep apnea
c. Identifying airway abnormalities including subglottic stenosis
2. Endotracheal intubation
a. Choosing endotracheal tube size
b. Confirming endotracheal intubation
i. direct assessment with transtracheal visualization
ii. indirect assessment with lung sliding or diaphragmatic movement
3. Assessing depth of endotracheal tube placement
a. Cuff placement
b. Tip of the endotracheal tube placement
4. Identification of the site for cricothyroidotomy
5. Miscellaneous applications
a. One lung ventilation

An online search of PubMed and Google Scholar was performed to identify appropriate manuscripts for inclusion using a combination of the following key words: ultrasound, airway, endotracheal intubation, pediatric anesthesiology, pediatrics, cricothyrotomy, and cricothyroidotomy. The subsequent review included case reports and case series, retrospective and prospective studies, systematic reviews and meta-analyses, clinical guidelines, and other narrative reviews. The literature search was restricted to studies published in English. The authors who have clinical experience in anesthesiology, pediatric anesthesiology, and pediatric critical care medicine reviewed the articles and decided which studies to include for the review by consensus, with a specific focus on relevant pediatric anesthesiology and pediatric critical care articles as well as illustrative articles from the adult literature. These specific adult studies were included if they focused on novel aspects of airway ultrasound, which may have applications in pediatric-aged patients, but which have not been specifically performed in that age group. Systematic reviews and meta-analyses were preferentially selected, followed sequentially by randomized controlled trials, prospective studies, retrospective studies, case reports, and other narrative reviews when alternate data were not available. Additionally, the reference list of included articles was reviewed and additional published manuscripts identified.

## Airway Assessment

Endotracheal intubation remains a primary tool for airway management in various clinical scenarios in operating rooms, intensive care units, and emergency departments [6]. Traditionally, airway assessment includes the history of previous endotracheal intubations, assessment of cervical spine mobility, and an appraisal of head, neck, and intra-oral anatomy [7]. Endotracheal intubation and its depth are confirmed by direct visualization during laryngoscopy, chest auscultation, capnography, and a chest radiograph. Due to its superior diagnostic accuracy, portability, familiarity, accessibility, safety, and non-invasive nature, POCUS has emerged as an important tool for airway assessment and management [4, 8, 9]. Reported applications of airway ultrasound include assessment of the airway prior to endotracheal intubation to identify patients with an unanticipated difficult airway. POCUS can also serve to confirm ETT placement particularly during resuscitation in a patient with cardiac arrest where capnography can be unreliable. Airway ultrasound has also been used to rule out esophageal or main stem intubation and to identify the CTM prior to cricothyrotomy in the “cannot intubate – cannot ventilate” situations [10–13].

## Sono-Anatomy of the Upper Airway

**Figure 1:**
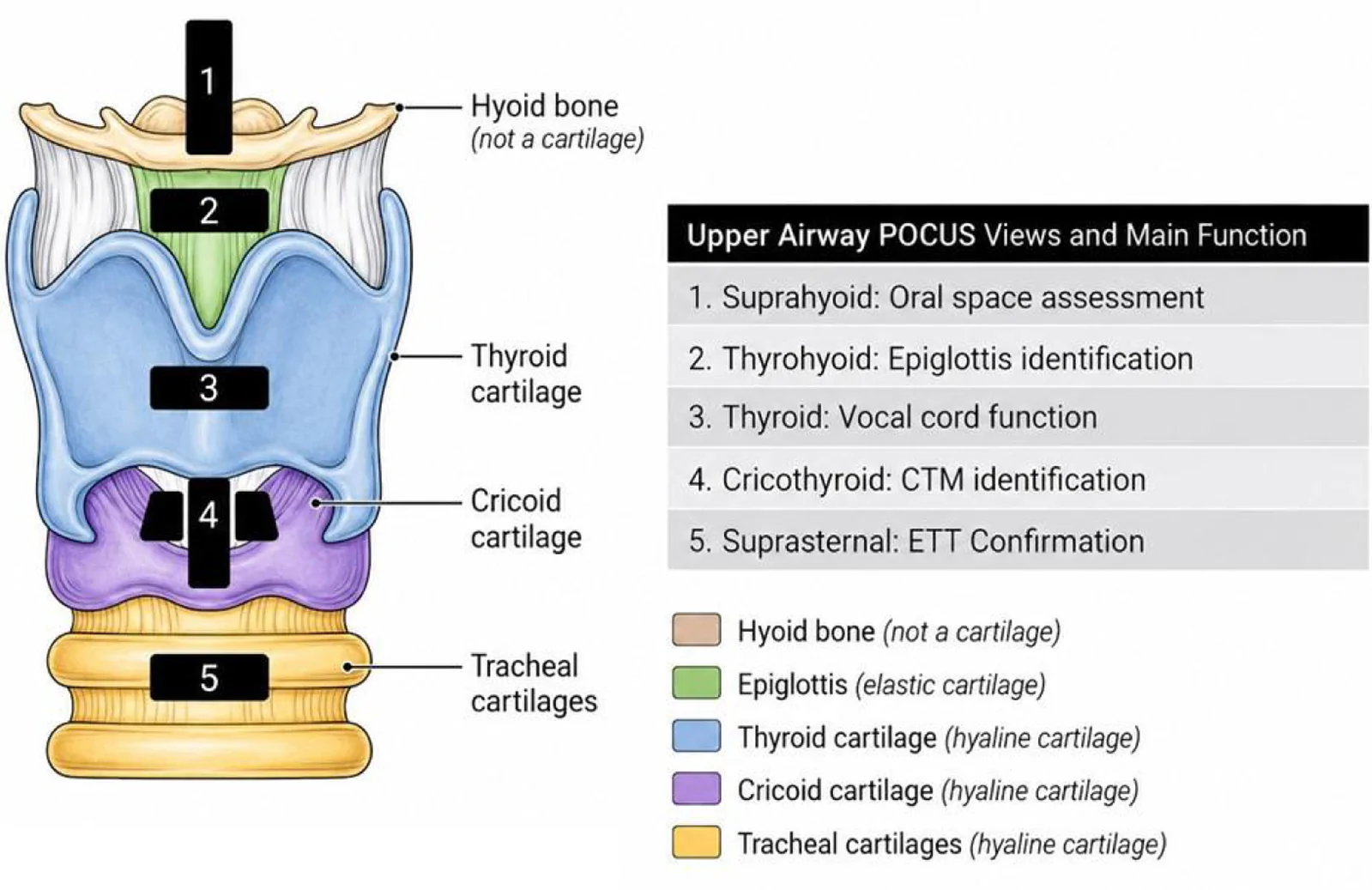
Ultrasound of the airway can be performed using transverse, sagittal and parasagittal views at multiple levels including the suprahyoid, thyrohyoid, thyroid, cricothyroid, and suprasternal levels, allowing detailed visualization of airway structures. CTM: cricothyroid membrane; ETT: endotracheal tube; POCUS: point-of-care ultrasound.

Ultrasound of the airway can be performed using transverse, sagittal, and parasagittal views at multiple levels including the suprahyoid, thyrohyoid, thyroid, cricothyroid, and suprasternal levels, allowing detailed visualization of airway structures (Fig. 1). Both the linear and the curvilinear probes are used for visualization of the upper airway. The linear probe has been shown to be useful in visualizing the superficial structures of the airway which include the epiglottis, vocal cords, and the CTM [4, 5]. The curvilinear probe is better suited for visualization of deeper structures such as the base of tongue and the esophagus. Specific measurements, such as the thyromental distance, may also use the curvilinear probe due to its larger footprint.

**Figure 2:**
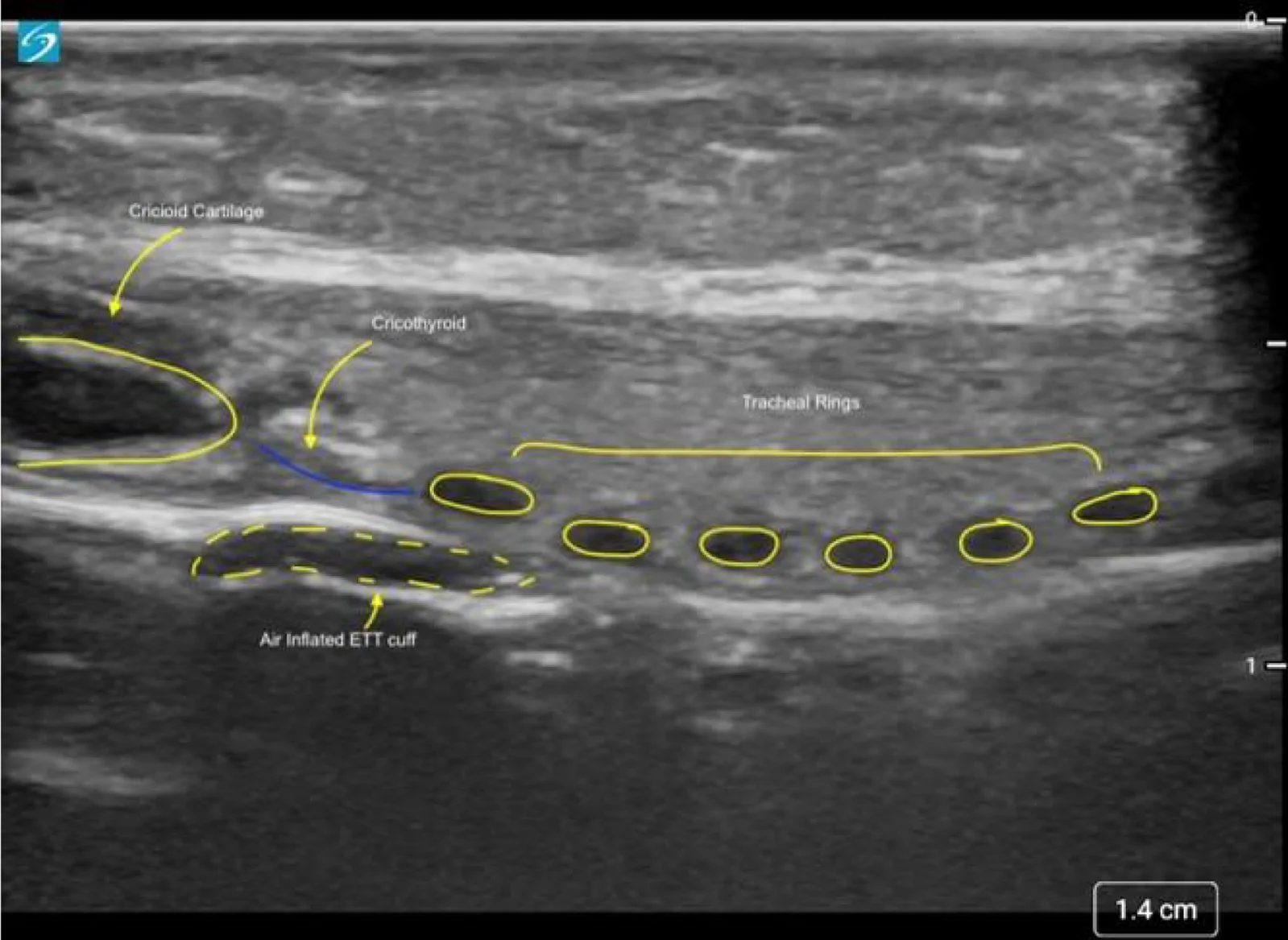
Sagittal ultrasound view of the airway showing the cricoid cartilage to the left at the top of the airway, the cricothyroid membrane (blue line), and tracheal rings. Air can be seen in the inflated endotracheal tube (ETT) cuff.

The transverse and the sagittal views are usually sufficient for airway visualization (Fig. 2). The parasagittal axis can be used to aid in better visualization of midline structures such as the CTM during cricothyroidotomy. Scanning is done with the patient in a supine position with the neck in neutral, ramped, or hyperextended position. Hyperextension allows for increased surface area for the probe footprint and may improve visualization of structures. Patient maneuvers can be utilized to better elucidate airway anatomy. Having the patient swallow may aid in the visualization of the esophagus and having the patient phonate can help to evaluate vocal cord function [14].

## Ultrasound to Evaluate Laryngoscopy and the Difficult Airway

### Adult investigations

The incidence of difficult airway and difficult endotracheal intubation varies from 5% to 22%, depending on the definition and patient population studied. Although relatively uncommon, these events can lead to significant morbidity or mortality if not anticipated [15–17]. Importantly, even patients classified preoperatively as having an “easy airway” may prove difficult to intubate or ventilate [16–18]. Conventional airway assessment relies on bedside physical examination tools such as the modified Mallampati score, upper lip bite test, mouth opening, and thyromental distance. Although widely used, these tools have limited sensitivity and specificity, reducing their reliability and applicability as screening tests [19, 20]. Composite scoring systems such as the Simplified Predictive Intubation Difficulty Score (SPIDS) and Total Airway Score (TAS) were developed to improve prediction by incorporating multiple clinical parameters. Although these composite scores may improve discrimination compared with individual airway measurements, their predictive performance remains variable across patient populations [21, 22].

Ultrasound, with its wide availability and non-invasive nature, is readily tolerated by patients and may have a role as an adjunct to these clinical airway assessment tools. POCUS may be able to complement traditional physical examination by providing real-time visualization and measurement of key oropharyngeal and tracheal structures including the epiglottis, tongue, hyoid, and vocal cords that previously required radiographic imaging [23]. Rapid ultrasound assessment may identify difficult laryngoscopy using ultrasound measurements of anterior neck soft tissue thickness, evaluation of tongue size, oral cavity space, and laryngeal position. Ultrasound derived measurements can be precise, comparable with other radiologic imaging techniques, presenting unique opportunities for measuring relevant pathology and structures in the airways [24].

A meta-analysis of adult studies by Bhargava et al evaluated sonographic predictors of difficult direct laryngoscopy in adults across three principal domains or measurements [25]. The three domains included the anterior neck tissue thickness domain (TTD), anatomic position domain (APD), and the oral cavity space domain (OSD). The anterior TTD, which includes distance from the skin to epiglottis (DSE), demonstrated a pooled sensitivity of 76% and specificity of 77%. The APD, assessed primarily through assessment of the hyo-mental distance (HMD) in the neutral position, showed the strongest predictive accuracy, with a specificity of 86% and a sensitivity of 74%. In contrast, the OSD, comprising tongue thickness and oral cavity height, offered a lower pooled sensitivity of 53%, but retained moderate specificity (77%).

Subsequent studies have reinforced these findings. Carsetti et al evaluated ultrasound-based predictors of difficult direct laryngoscopy in adult patients undergoing elective surgery [15]. The study confirmed the diagnostic value of DSE, skin to hyoid bone distance (DSHB), skin to vocal cord distance (DSVC), and ratio of pre-epiglottic space to the epiglottis-to-vocal cords distance. While all parameters differed significantly between easy versus difficult direct laryngoscopy, DSE emerged as the most reliable and widely adopted metric associated with difficult direct laryngoscopy. The predictive value of DSE for difficult direct laryngoscopy in various ethnicities and patient populations is well supported by other studies [9, 24–29].

When comparing and evaluating these studies, identifying which specific ultrasonographic measurement is the best predictor of difficult direct laryngoscopy and endotracheal intubation is essential. Evaluating the OSD, Ohri et al used two-dimensional ultrasonography to calculate tongue volume in real time and correlate it with the modified Cormack-Lehane grading observed during direct laryngoscopy [23]. A larger tongue volume correlated with a higher incidence of difficulties with direct laryngoscopy and endotracheal intubation. These findings correlate with the clinical assumption that size and volume of tongue affect direct laryngoscopy and view of the glottis due to limitations of the submandibular space. HMD has been shown to be the best predictor of difficult laryngoscopy and difficult endotracheal intubation within the APD [25]. Furthermore, Gomes et al identified HMD as the most consistent predictor overall, whereas tongue thickness was found to be the most reproducible measure within the OSD [9].

Lin et al in their comprehensive review on the utility of POCUS in airway management proposed the Difficult Airway Evaluation with Sonography (DARES) protocol to unify these approaches, which integrated validated parameters across domains to enhance standardization and risk stratification [14]. These sonographic measurements offer objective, quantifiable data that enhance the prediction of difficult direct laryngoscopy with greater precision and consistency than traditional bedside physical examination alone.

### Applications in pediatric-aged patients

**Figure 3:**
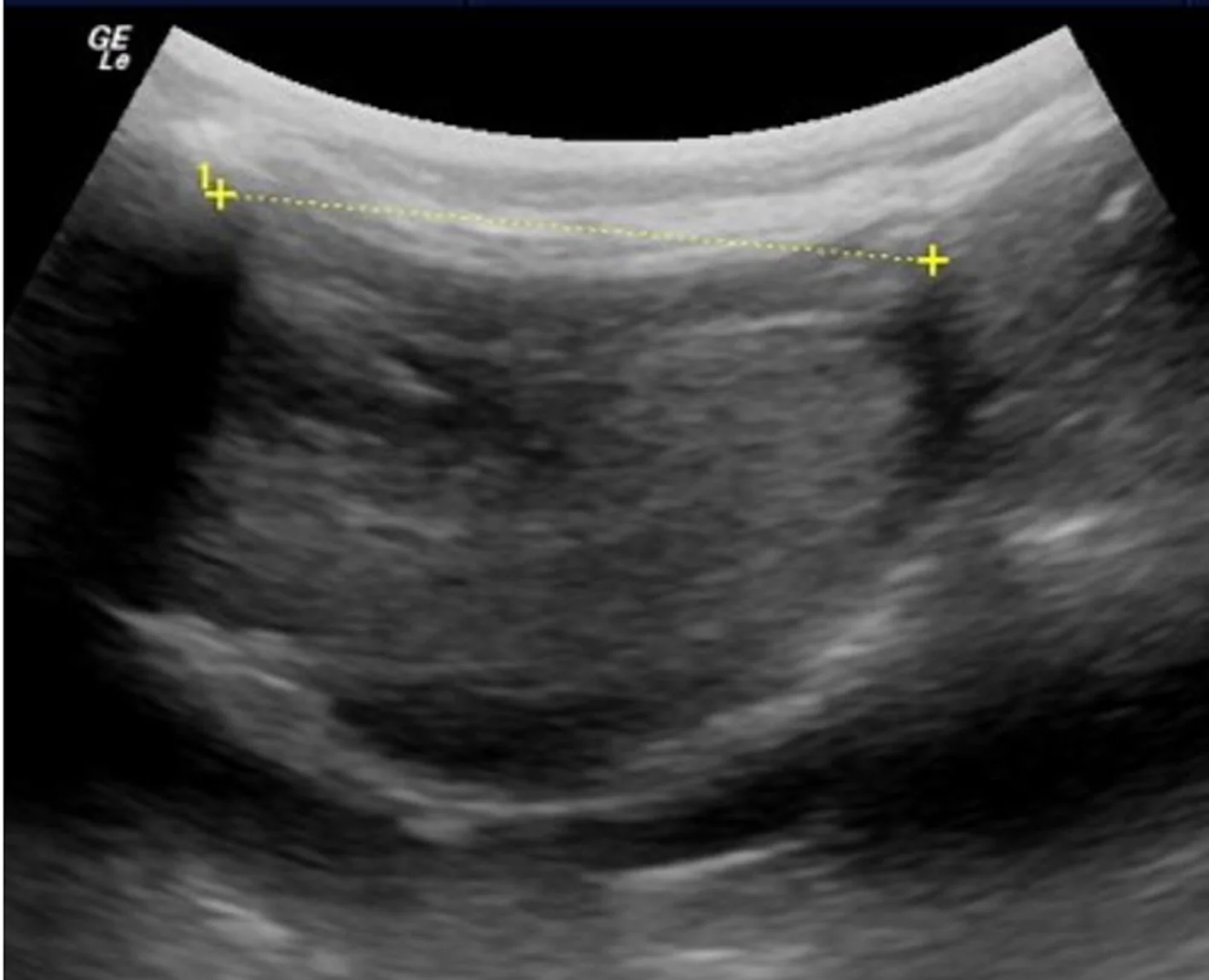
Airway ultrasound in children with measurement of the hyo-mental distance (HMD). For this measurement, the probe is placed in the sagittal plane in the submental area.

**Figure 4:**
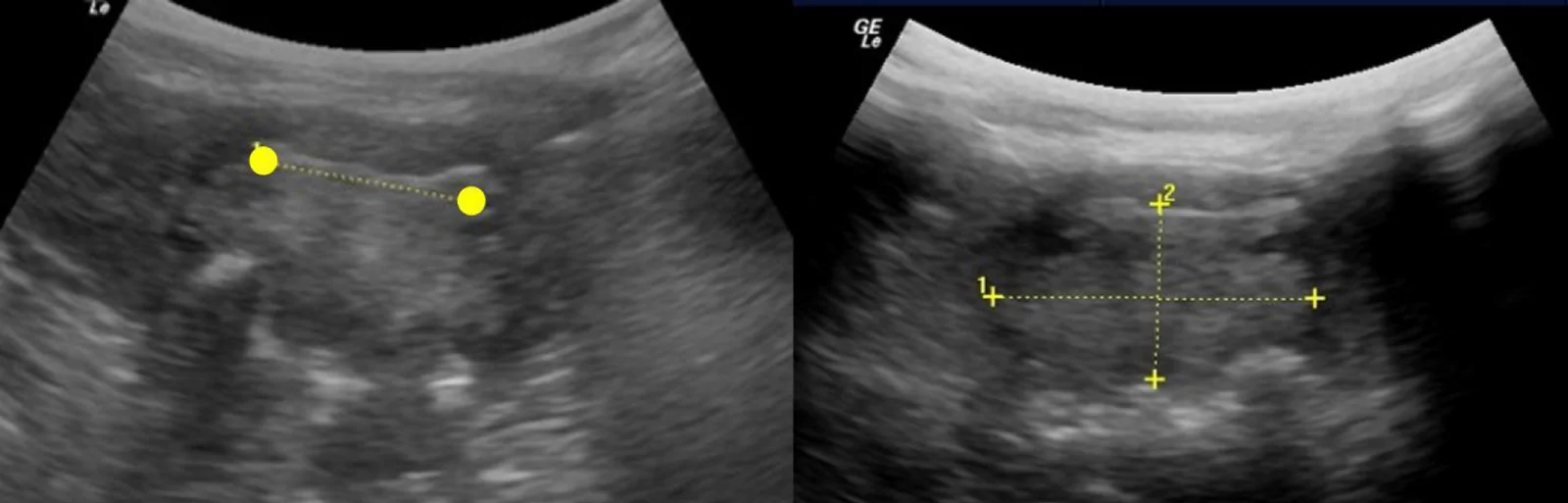
Oropharyngeal ultrasound in children. Measurement of the distance between the lingual arteries at the base of the tongue (left) and measurement of the width (1) and height (2) of the tongue at its base at the back of the oropharynx (right).

The application of POCUS for airway assessment in children is an evolving field, with several sonographic parameters demonstrating potential utility as predictors of difficult direct laryngoscopy and endotracheal intubation. While the literature is still developing and specific conclusions may be limited by heterogeneity in study design and patient populations, key measurements have emerged as promising tools in pediatric airway evaluation. Among pediatric predictors, the hyo-mental distance ratio (HMDR), calculated as the HMD at maximum head extension divided by the HMD in the neutral head position, has shown promising predictive value. In children less than 2 years of age, an HMDR cutoff of approximately 1.055 predicted difficult direct laryngoscopy with a sensitivity of 100% and specificity of 86.9%, while in children 5–12 years of age, a shortened hyo-mental distance in extension (HMDE) was associated with difficult laryngoscopy [30, 31]. Consistent with adult data, an increased DSE in children has been shown to correlate with a more anterior epiglottis and reduced glottic exposure [31]. Tongue-related POCUS measurements, including tongue thickness (TT), TT/oral cavity height (TT/OCH), and TT/thyromental distance (TT/TMD) ratios, have demonstrated potential, although their applicability may be limited by variability across age groups (Figs. 3 and 4) [14, 32].

Finally, sonographic parameters in children targeting proximal airway landmarks, such as the epiglottis and hyoid, may offer superior diagnostic accuracy compared with those measured near distal structures like the CTM or vocal cords [32, 33]. These studies seem to support the potential clinical utility of proximal ultrasound markers in predicting difficult direct laryngoscopy in children.

In pediatric patients, especially infants and toddlers, standard airway examination and other conventional airway assessment tools (e.g., Mallampati classification) may be impractical due to lack of cooperation and limited sensitivity and specificity when compared with the adult population. This makes accurate preoperative airway assessment using clinical and physical assessment tools particularly challenging in children. The non-invasive and objective nature of ultrasound makes it ideal for pediatric airway assessment in young patients, encouraging future clinical research and endeavors in this area [34].

## Grading or Identification of Obstructive Sleep Apnea (OSA)

POCUS has emerged as a potential tool for identification and grading of patients with previously undiagnosed OSA [35, 36]. Within this context, ultrasonography has the benefit of not requiring sedation or anesthesia for completion of an exam, thereby offering advantages over computed tomography (CT) or magnetic resonance imaging (MRI) in infants and younger children. Ultrasonography can be performed rapidly at the bedside without need for prior planning or scheduling. Unlike CT imaging, there is no radiation exposure and unlike MRI, no concerns of ferromagnetic materials or other MRI associated safety concerns.

Sleep disordered breathing (SDB) includes a clinical disease spectrum ranging from snoring and upper airway resistance to a complete upper airway obstruction or OSA. The prevalence of SDB in children ranges from 9% to 12%, while that of OSA is 1–3% [37, 38]. Traditionally, the severity of OSA has been described in terms of the apnea-hypopnea index (AHI) with classification into mild (5–15/h), moderate (15–30/h), and severe (> 30/h) based on the number of events per hour. Although polysomnography remains the gold standard in the diagnosis of OSA, its widespread use is limited by cost, accessibility, and the need for overnight hospitalization. In addition, sleep studies are time-consuming, and may result in missed school or work days, creating further inconvenience for patients and families. Commonly used questionnaire-based screening tools offer a more accessible alternative, but they may lack specificity and have a poor negative predictive value [39, 40]. A recent systematic review and meta-analysis evaluating POCUS in adults identified several parameters, including distance between lingual arteries, resting tongue thickness, and tongue base thickness during Muller maneuver, all of which correlated moderately with moderate-severe OSA [35]. Additionally, the non-airway parameter of carotid intimal media thickness was identified as having a low to moderate correlation with moderate to severe OSA in adults.

**Figure 5:**
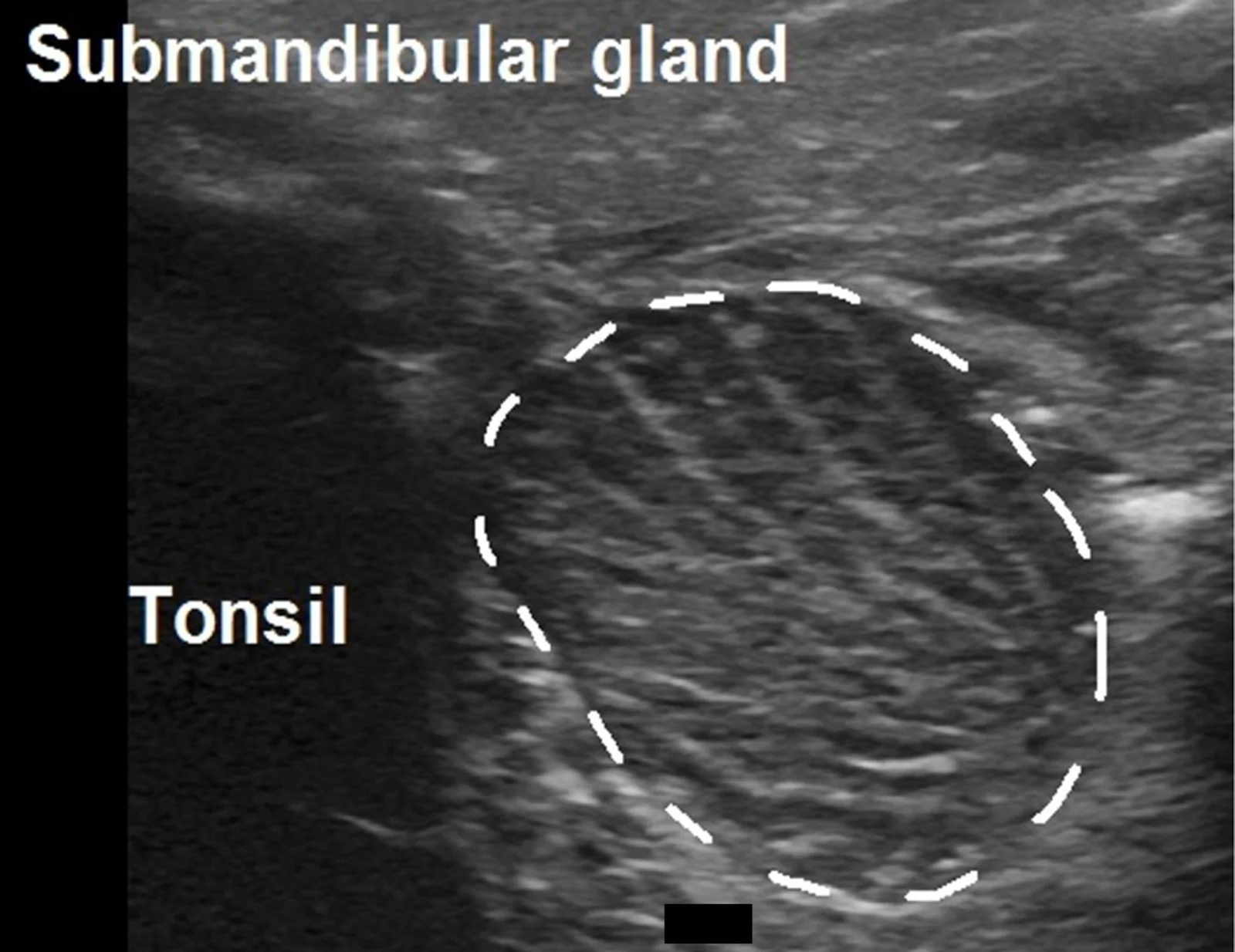
Submandibular ultrasound showing identification of tonsillar size.

Burns et al in a systematic review of 12 studies (1,237 pediatric patients) evaluated the role of surface ultrasound as a POCUS modality for OSA screening in pediatric-aged patients [36]. The review examined both airway-related and non-airway sonographic parameters and categorized the studies into two major groups based on the nature of their association with OSA severity and diagnosis. One group of parameters directly correlated with polysomnographic indices including AHI, respiratory-disturbance index (RDI), and mean oxygen saturation. The authors reported that lateral pharyngeal wall thickness, total retropharyngeal neck thickness, carotid intima–media thickness (cIMT), and carotid distensibility showed meaningful associations with disease severity. The second group examined indirect relationships, primarily focusing on adenotonsillar anatomy (tonsillar dimensions and adenoid thickness). Importantly, tonsillar volume alone did not reliably correlate with OSA diagnosis or severity; instead, these metrics were validated against surrogate reference standards (surgical specimen volume, radiography, and nasopharyngoscopy). Figures 4 and 5 demonstrate examples of the use of ultrasonography for airway imaging in children with measurement of the distance between the lingual arteries, height and width of the tongue, and direct ultrasonography of the tonsil.

Lin et al in a study cohort of 82 pediatric patients demonstrated positive correlation between OSA and lateral pharyngeal wall thickness (LPW) during both the Muller maneuver and at rest [41]. There was also a positive correlation between OSA and total neck thickness. However, there was no correlation between AHI and tonsil volume. Au et al in two studies demonstrated an association between LPW and OSA (AHI > 1) (odds ratio (OR) = 1.73; 95% confidence interval (CI), 1.05–2.83), when adjusted for age, sex, body mass index (BMI), and Z-score [42, 43]. The association with moderate to severe OSA was more significant (OR = 3.65 95%; CI, 1.53–8.68). Collectively, these findings underscore the potential of airway ultrasound while highlighting the need for standardized pediatric protocols.

Tonsillar size is the most frequently evaluated airway parameter despite the absence of a consistent direct correlation with OSA severity. Although pediatric OSA guidelines emphasize tonsillar hypertrophy, relatively few studies have examined its relationship with objective measures such as the AHI. Available evidence suggests a moderate to strong correlation between tonsillar size assessed by ultrasound with either intraoperative findings or excised surgical specimens, supporting the validity of imaging-based tonsillar size assessment. While current guidelines support tonsillectomy for children with OSA confirmed on overnight polysomnography, tonsillar hypertrophy represents only one component of upper-airway obstruction [44]. Additional soft-tissue structures and craniofacial skeletal features may play an important role in determining airway patency and contribute to the multifactorial etiology of pediatric OSA, which may be better assessed in a dynamic real-time fashion using airway ultrasonography [45].

Regarding non-airway parameters, several studies have examined the association between long standing OSA and vascular markers (cIMT and carotid distensibility) assessed using ultrasound [46–48]. While the relationship between OSA and these vascular parameters has been well documented in adult populations with cardiovascular disease, findings in the pediatric population have been inconsistent. In pediatric studies, Iannuzzi et al, Marshall et al, and Tagetti et al reported no significant association or correlation between OSA and cIMT [49–51]. Tagetti et al demonstrated no correlation between cIMT and OSA severity, as assessed by the AHI, nor with other respiratory indices including oxygen saturation, RDI, or oxygen desaturation index [51]. However a weak, but statistically significant association was identified between carotid distensibility of the internal carotid artery and AHI (P = 0.03), suggesting that functional vascular alterations may precede detectable structural changes in children with OSA.

When compared with adults, relatively few pediatric studies have directly evaluated ultrasound-derived measures, whether vascular or airway-related, and their correlation to OSA. One of the challenges in this area when focusing on the pediatric-aged patient remains the variability in the association of various physical features (BMI, tonsillar size, neck circumference) with OSA and AHI. These factors highlight the heterogeneity of OSA in pediatric-aged patients especially the pre-pubertal group where there are marked differences between etiology and presentation when compared to adults or even adolescents. These variabilities identify important gaps in understanding the structural and functional correlations of pediatric OSA.

## Identifying Airway Abnormalities Including Subglottic Stenosis

**Figure 6:**
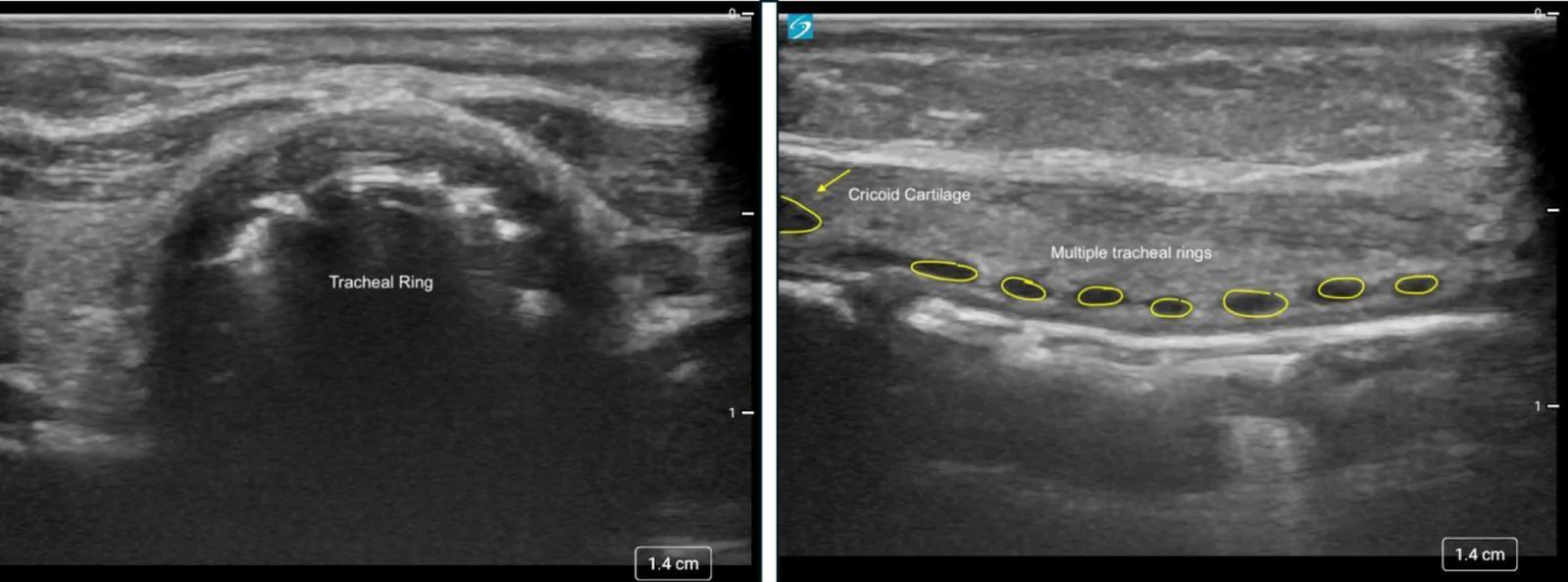
Transverse (left) and sagittal (right) ultrasound views of the area demonstrating the trachea, subglottic area, and cricothyroid membrane.

Various studies have evaluated the use of ultrasound to visualize the trachea and subglottic area (Fig. 6). Giguere et al in one of the earliest ultrasound studies compared US and video bronchoscopy to direct caliper measurements of the dissected larynx in an animal model, demonstrating agreement of the two methods in measuring the subglottic lumen diameter with a CI of 95%, thus validating ultrasound as a potential tool for measuring the subglottic lumen [52]. Miles described the use of ultrasound for assessment of vocal cord movement while Kundra et al demonstrated the utility of airway ultrasonography for diagnosing superior laryngeal nerve and recurrent laryngeal nerve injuries after thyroid and parathyroid surgery in adults [53, 54].

Garel et al described the use of trans-laryngeal ultrasound for evaluating upper-airway pathology in infants and children [55]. In this early feasibility study of 33 patients (1 day to 15 years of age), ultrasound effectively identified several functional abnormalities, including vocal cord paralysis, using dynamic maneuvers such as deep breathing and phonation. The authors also demonstrated the utility of ultrasound in detecting pathologic lesions, including subglottic masses and subglottic stenosis. For patients with subglottic stenosis, ultrasound allowed visualization of arytenoid motion and identification of anterior synechiae when present, highlighting its potential complementary role to airway endoscopy. Bisetti et al reported laryngeal ultrasound as a safe and accurate tool in diagnosing vocal cord pathology including hemangiomas, laryngeal stenosis, and vocal cord paralysis [56]. The study compared the results with flexible fiberoptic laryngoscopy and suggested that ultrasound could be used as an effective supplemental tool particularly in children with a limited ability to cooperate with a more invasive examination.

A prospective, double-blinded pediatric clinical study compared ultrasonography, video bronchoscopy, and ETT sizing for measurement of subglottic airway diameter [57]. Ultrasonographic measurements showed a strong correlation with both video-bronchoscopy and ETT sizing; however, ultrasound consistently underestimated absolute subglottic dimensions. Despite the authors being less enthusiastic about use of ultrasound for absolute airway sizing or ETT selection, they opined that it may serve as a useful non-invasive tool for longitudinal monitoring of changes in subglottic caliber over time. The study did not include children with established subglottic stenosis, which may have limited their conclusions regarding diagnostic performance of US in airways with pathologic narrowing.

Bell et al used ultrasonography to identify extraluminal stents as well as glottic, subglottic, and tracheal pathology in four patients who had previously undergone tracheal reconstruction [58]. US findings were compared with endoscopic images obtained during direct laryngoscopy and bronchoscopy. Subglottic measurements obtained using ultrasound were within 0.1–0.5 mm of the outer diameter of the appropriate-sized ETTs. They concluded that ultrasound can be used as a surveillance tool in patients who would require repeated laryngoscopy or other imaging modalities, thus avoiding anesthesia and radiation exposure. However, they cautioned that ultrasound did not visualize tracheotomy tubes or posterolateral pathology. Recent work in adults has suggested that the improved accuracy of airway evaluations using 3D reconstructions with ultrasound may allow better assessment of airway anatomy including the anteroposterior diameter which is challenging to visualize using conventional ultrasound. The preliminary results have been shown to correlate well with MRI assessment in adults [59].

## Endotracheal Intubation and Cuff Placement

### ETT cuff position and depth of insertion

**Figure 7:**
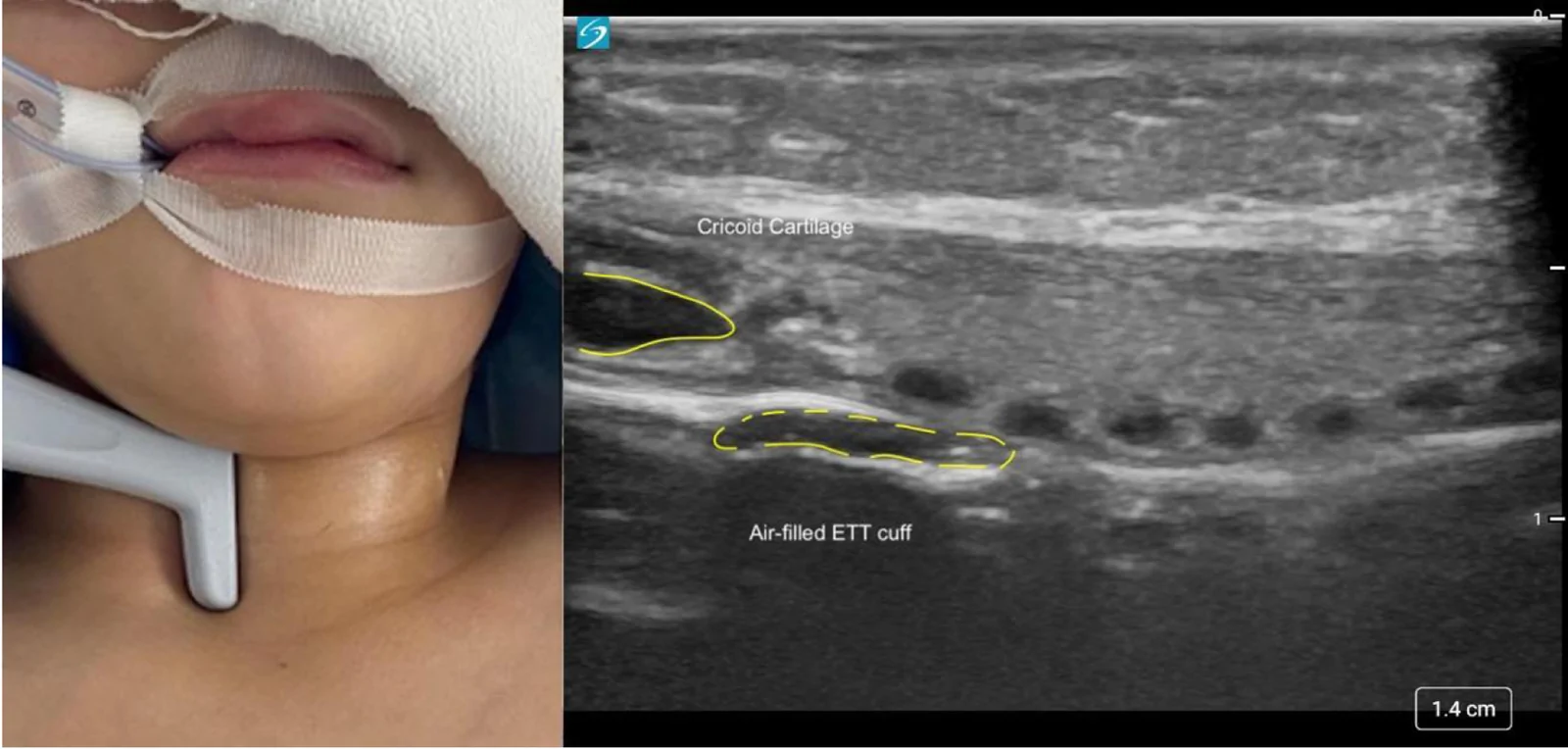
High-frequency linear probe placed on the anterior neck in the para-sagittal, long-axis plane (left) for airway ultrasonography. The cricoid cartilage and tracheal rings appear as anechoic structures. The anterior row of tracheal cartilages (anechoic structures) in the longitudinal plane is sometimes referred to as a “string of beads.” The air-filled cuff of the endotracheal tube also appears as an anechoic structure deep to the tracheal cartilages in a two-dimensional plane.

**Figure 8:**
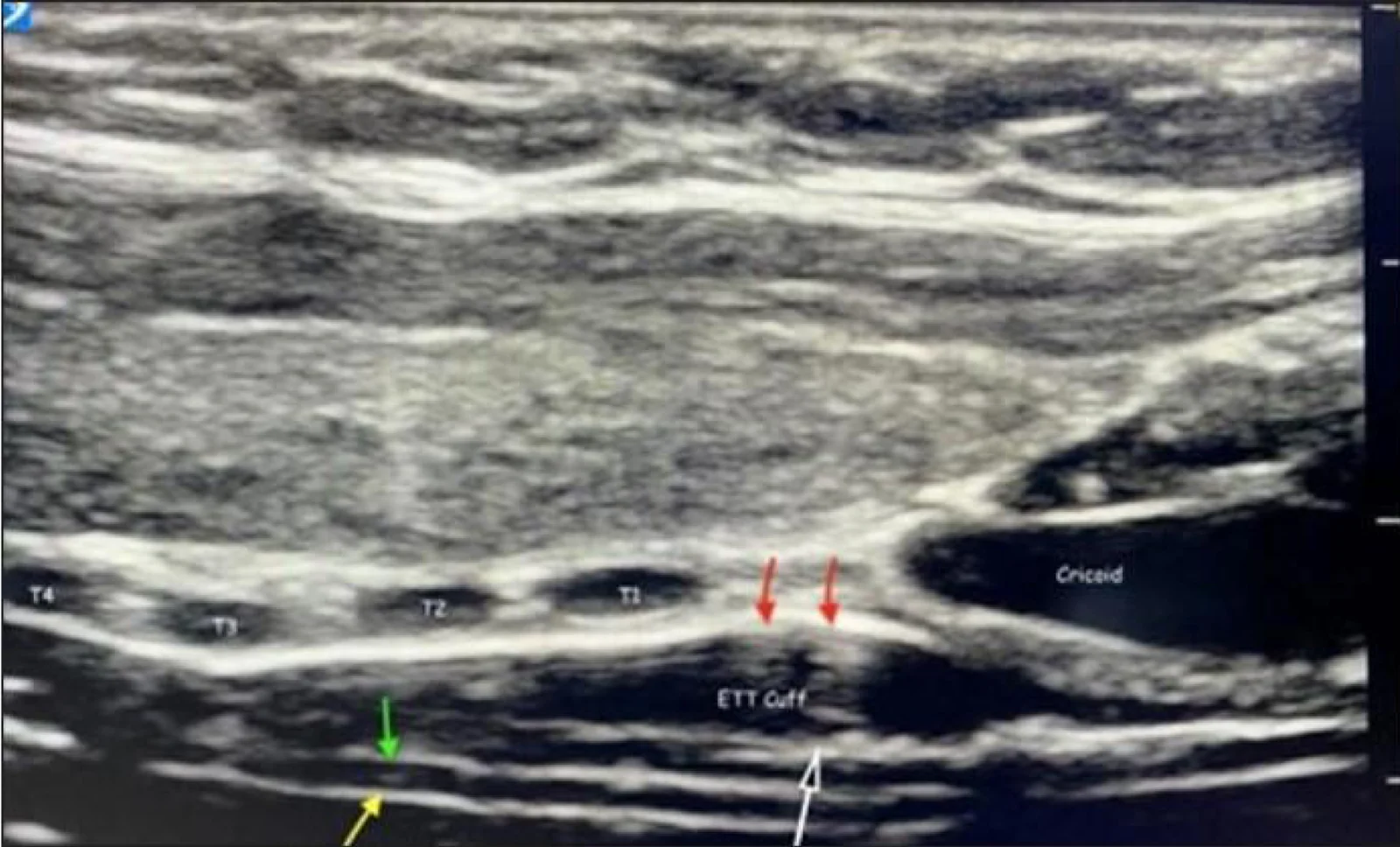
Ultrasonographic images of the airway and endotracheal tube (ETT). The cricoid is an anechoic structure to the right and the tracheal rings are numbered (T1 to T4). To enhance visibility on ultrasound, the cuff of the ETT is filled with saline (white arrow). The cephalad aspect of the cuff is positioned at the lower aspect of the cricoid. A linear hyperechoic line (red arrows) on the longitudinal plane of the trachea is formed by reverberation artifacts from the air-mucosa interface within the trachea (red arrows). The walls of the ETT within the trachea are seen as two hyperechoic lines (yellow and green arrows).

Confirming the depth and position of the ETT cuff is imperative in reducing adverse effects related to endotracheal intubation and cuff inflation such as laryngeal injury, subglottic stenosis, and inadvertent tracheal extubation. The cricoid cartilage is defined as the narrowest rigid aspect of the pediatric airway. If the ETT cuff lies at the cricoid, mucosal ischemia and ulceration may occur, leading to post-extubation stridor or subglottic stenosis. Positioning the cuff entirely below the cricoid cartilage against the more distensible tracheal rings is generally recommended as a means of limiting airway trauma [60]. When a high-frequency linear probe is placed on the anterior neck in the sagittal or para-sagittal long-axis plane, the cricoid cartilage and tracheal rings appear as anechoic structures in the two dimensional image (Fig. 7). A linear hyperechoic line seen posteriorly on the longitudinal plane of the trachea is formed by reverberation artifacts from the air-mucosal interface of the cuff against the tracheal mucosa. A saline-inflated cuff is seen as a hyperechoic band with posterior shadowing (Fig. 8). By confirming that the cuff is distal to the cricoid, clinicians can safely adjust ETT depth in real time [5, 61].

In a prospective, non-randomized study of 80 pediatric-aged patients aged 1–78 months, ultrasound was used to locate the ETT cuff relative to the cricoid and tracheal rings [61]. The cephalad end of the ETT cuff was found at the level of the cricoid in 16.3% of patients, at the first, second, and third tracheal rings in 27.5%, 23.8%, and 17.5% patients, respectively, and at or below the fourth tracheal ring in 15% of patients. The authors suggested that this observed inconsistency highlights known variability in pediatric airway anatomy, underscoring the value of real-time ultrasound imaging. These findings align with anatomical data obtained from an *in vitro* dissection study of pediatric larynxes, which demonstrated that the cuff location on modern ETTs frequently overlaps the cricoid cartilage rather than remaining entirely distal to it [62]. With the currently available ETTs, especially in the smaller sizes, the proximal portion of the cuff may impinge upon the cricoid. The cuff malposition may lead to mucosal ischemia and subsequent subglottic injury. Based on these observations, the authors advocated for a redesign of cuff position on the ETT by current manufacturers. Together, both ultrasound evidence *in vivo* and anatomical data *in vitro* reinforce the principle that optimal cuff position below the cricoid cartilage may not be achieved in a significant percentage of patients. This gap further strengthens the role of POCUS as a real-time, non-invasive tool for confirming and correcting cuff depth to minimize airway morbidity while avoiding the radiation related to radiographic imaging.

Recent evidence has expanded the role of POCUS for confirming the depth of ETT placement and as a means of avoiding mainstem intubation. The tracheal carina cannot be visualized directly with ultrasound because air-filled lungs create acoustic artifacts that obscure the structure. Therefore, adjacent vascular landmarks, most commonly the right pulmonary artery or the apex of the aortic arch are used as reliable sonographic surrogates for estimating the position of the carina. Levkovitz et al demonstrated a strong correlation between the ultrasound distance from the ETT to the right pulmonary artery (RPA) and the ETT to carina distance on standard chest radiographs, supporting the use of the RPA as a surrogate anatomical landmark for the carina [63]. Consequently, positioning the tip of the ETT 0.5–1.5 cm above the RPA provides a reasonable estimate for mid-tracheal placement when the head is in a neutral position. The accuracy of this technique was confirmed by Salvadori et al, who demonstrated that using the superior margin of the RPA as a landmark, is as reliable as a chest radiograph, but significantly faster with a median performance time for ultrasound of 3.2 min [64].

Using the RPA as a reliable surrogate marker for the carina, a systematic review and meta-analysis by Congedi et al evaluated 602 neonatal endotracheal intubations across 14 studies [65]. The authors specifically evaluated the capability of POCUS to determine the correct ETT insertion depth. Across the studies, the ETT tip was most closely referenced to the superior aspect of the RPA or the apex of the aortic arch, both serving as reliable surrogates for the carina. POCUS identified the ETT in 96.8% of cases, with a sensitivity of 93.4% for detecting an appropriately positioned tip compared with chest radiography. The RPA landmark yielded the highest sensitivity (approaching 100%), whereas the aortic arch marker showed slightly lower accuracy. Although specificity remained limited due to the relative paucity of mal-positioned ETTs, these findings highlight that ultrasound can provide real-time, radiation-free localization of the ETT tip in neonates, allowing clinicians to detect ETTs that are positioned too high or too low within the trachea.

A transverse ultrasound scan at the suprasternal notch has been evaluated as a surrogate marker of ETT depth when a fluid-filled cuff is used in older infants and children. A prospective study in 75 pediatric patients demonstrated that ultrasound visualization of a saline-inflated cuff with the probe placed at the level of the suprasternal notch correlated with fluoroscopic confirmation of correct ETT tip position in 95% of cases, with the tip reliably located below the clavicles and at least 1 cm above the carina [66]. This simple and reproducible technique using the acoustic window of the suprasternal notch helps avoid radiation exposure, and provides real-time confirmation of ETT placement, making it a practical, bedside tool to complement standard depth assessment.

### Choosing correct ETT size

An appropriately sized ETT is critical for effective ventilation, minimizing airway trauma, and prevention of complications such as post-extubation stridor and subglottic stenosis. POCUS has emerged as a potential tool for assessment of ETT size in real time. Liu et al in their systematic review analyzed six RCTs and 16 diagnostic studies involving 1,816 pediatric patients for whom ultrasound was used for ETT size measurement and selection [67]. The ultrasound exam was performed by placing the linear probe on the anterior aspect of the neck in the midline and measuring the minimal transverse diameter of the subglottic airway at the level of the cricoid. The diagnostic accuracy of the studies varied somewhat, but in general, airway ultrasound could be used to determine ETT size, confirm endotracheal intubation, and document ETT position with a clinically useful degree of diagnostic accuracy. When specifically considering the accuracy of airway ultrasound in predicting ETT size, US was consistently better than traditional methods, such as height formula, age formula, and the width of the little finger with the majority of the studies demonstrating an accuracy ≥ 90%.

Singh et al in a prospective study of 100 patients (12–60 months of age) demonstrated that US was the most sensitive (100%) method in predicting the appropriate ETT size [68]. Tracheal diameter was measured as the transverse air column diameter at the cephalad end of the cricoid cartilage which is narrower than the caudal part. The authors judged their ETT size selection as “best fit” if the air leak was satisfactory at 15–20 cm H_2_O airway pressure, upsizing 0.5 mm if the leak occurred at a pressure < 10 cm of H_2_O and downsizing by 0.5 mm if resistance was met or no leak was detected. Using Pearson’s correlation, the authors demonstrated that ultrasound correlated more strongly with actual ETT size (r = 0.943) than alternative methods including age-based formula (r = 0.743), body length-based formula (r = 0.683), finger-based formula (r = 0.587), and multivariate formula (r = 0.741).

Similarly, Hao et al evaluated a cohort of children with congenital scoliosis and found a strong correlation between best-fit ETT size and the ETT size predicted by ultrasound, particularly in patients with thoracic and lumbar scoliosis [69]. Kim et al demonstrated a strong correlation between ultrasound measured subglottic diameter and the actual outer diameter of the ETT (ETT_OD_). Using these data, they developed an empirical equation with the ETT_OD_ (mm) = (0.01 × age (months) + 0.02 × height (cm) + 3.3) for choosing an appropriate ETT in children ≥ 12 months of age [70]. The formula functions reliably across both cuffed and uncuffed ETT variations. These findings are reinforced by Shibasaki et al who evaluated patients 1 month to 6 years of age and reported a 98% rate of agreement for cuffed ETTs and 96% for uncuffed ETTs when using ultrasound to measure subglottic upper airway diameter [71].

Notably, none of the reviewed studies reported any protocol discontinuations due to adverse events and no specific adverse effects pertaining to ultrasound were noted, thereby highlighting the safety of this non-invasive technique. Ahn et al reported that pediatric patients tolerated ultrasound well which could result in fewer airway related adverse events [72]. They also reported a lower incidence of adverse events such as the need for ETT exchange, ETT cuff herniation, endobronchial intubation, and post-extubation stridor in patients where ultrasound was used for ETT selection. More recently, POCUS was used during the COVID-19 pandemic in pediatric patients to predict ETT size, thus helping avoid unnecessary airway manipulation and reducing the risk of occupational exposure to providers [67, 73]. Together, these studies demonstrate the safety and utility of using POCUS in determining ETT size as well as its potential advantages over traditional methods currently in place.

### Confirmation of endotracheal intubation

Accurate and rapid confirmation of correct ETT placement is a critical step in pediatric airway management. Clinical assessments including auscultation, chest excursion, and mist in the ETT have been used to document the intratracheal location of the ETT. Although quantitative capnography with a waveform is considered the reference standard for confirmation of ETT placement, its reliability may be impacted during low cardiac output states, cardiac arrest, or with lower tidal volume ventilations [74]. This is especially true in neonates and infants as small tidal volumes, rapid respiratory rates, and low pulmonary blood flow may impact the sensitivity of capnography [75].

POCUS has emerged as an increasingly important adjunct for confirmation of ETT placement in pediatric patients during various clinical scenarios. Ultrasound offers a rapid and non-invasive method for confirming ETT position and identifying common malpositions, while not being dependent on cardiac output or excretion of carbon dioxide. These advantages are particularly relevant in children who have smaller airway diameters, shorter tracheal lengths, and a higher risk of unrecognized esophageal or endobronchial intubation. Sonographic confirmation techniques may be broadly divided into direct methods, which visualize the ETT within the airway, and indirect methods, which infer correct placement based on lung ventilation or diaphragmatic movement [10, 12, 76, 77].

Direct ultrasound confirmation involves visualization of the ETT within the trachea using a high-frequency linear transducer placed transversely or longitudinally over the anterior neck, most commonly at the level of the suprasternal notch or CTM. The trachea appears as a hyperechoic air–mucosa interface with a posterior reverberation artifact, while the esophagus—when visualized—typically lies posterolateral to the trachea. Correct endotracheal intubation is characterized by visualization of a single air–mucosa interface, whereas esophageal intubation produces the characteristic double tract sign, in which a second hyperechoic air interface is visualized within the esophagus due to the presence of the ETT. This sign can be identified in real time during ETT insertion or immediately following endotracheal intubation and has been consistently described as a reliable indicator of esophageal intubation [11, 78, 79]. Transtracheal ultrasound allows confirmation of ETT location before initiation of ventilation, thereby reducing gastric insufflation and potentially, the risk of aspiration. In pediatric patients, the relatively superficial airway anatomy and thinner, soft tissues may facilitate visualization of tracheal structures; however, the smaller airway caliber and shorter tracheal length increase the risk of inadvertent endobronchial intubation, which cannot be reliably assessed using transtracheal ultrasound alone.

Marciniak et al demonstrated the clinical utility of airway ultrasound, showing its effectiveness for assessing ETT placement in pediatric anesthesia practice [80]. In a study of 30 pediatric patients (mean age of 48 months), real-time imaging was used to visualize passage of the ETT into the airway by visualization and identification of the trachea, tracheal rings, and the vocal cords. The authors noted widening of glottis as the ETT passed through the airway and correctly positioned the ETT by using lung sliding. A single case of esophageal intubation was readily recognized by visualization of the ETT in the esophagus in the left paratracheal space. The study documented the characteristic ultrasonographic findings of the pediatric airway during endotracheal intubation and provided preliminary evidence that ultrasonography may be useful for airway management in children.

Chou et al describing the tracheal rapid ultrasound exam (TRUE), demonstrated accurate differentiation between tracheal and esophageal intubation during emergency airway management in adults comparing its accuracy to ETCO_2_ monitoring [11]. The ultrasound probe was placed transversely on the neck in the suprasternal notch. Tracheal intubation was confirmed by identification of only one air–mucosa (A–M) interface with comet tail artifact and posterior shadowing while esophageal intubation was confirmed by two A–M interfaces (double tract sign). The median time to perform TRUE was 9 s and the overall accuracy was 98.2% with a high degree of correlation with capnography. Hoffmann et al further validated this approach, reporting high diagnostic accuracy with faster confirmation times compared with auscultation [78]. A systematic review and meta-analysis by Chen et al found that transtracheal US confirms endotracheal intubation in intensive care unit (ICU) patients with a pooled sensitivity and specificity exceeding 95% [81]. Although pediatric-specific data remain limited, preliminary clinical evidence supports the feasibility and diagnostic utility of this technique in children.

Transtracheal ultrasound is rapid, reproducible, and independent of pulmonary blood flow, making it particularly valuable in cardiac arrest, shock, or other clinical scenarios where the presence of ETCO_2_ may be reduced. However, it does not reliably assess ETT depth or detect mainstem bronchial intubation. Acoustic windows may be limited by obesity, subcutaneous emphysema, or distorted neck anatomy. Operator experience remains a key determinant of image acquisition and interpretation.

### Indirect assessment: diaphragmatic movement or lung sliding

Indirect ultrasound techniques confirm ETT placement by demonstrating effective lung ventilation or diaphragmatic motion following endotracheal intubation [82, 83]. These techniques are typically performed after mechanical ventilation has begun. Diaphragmatic ultrasound is relevant in pediatric patients, particularly neonates and infants, as the diaphragm plays a dominant role in their ventilation [82]. Ultrasound is performed via a subcostal or low intercostal window using a curvilinear probe to visualize the diaphragm. Correct endotracheal intubation and effective mechanical ventilation produce synchronous caudal diaphragmatic excursion while unilateral diaphragmatic movement may indicate endobronchial intubation [82, 83]. Kerrey et al compared lung ultrasound to chest radiography in 127 intubated pediatric patients in the emergency department [82]. The primary outcome was the agreement between the two modalities for ETT positioning. Chest radiography identified the ETT in the mainstem bronchus in 24 patients (19%), while there were no esophageal intubations. Ultrasound and chest radiography agreed on ETT placement in 106 patients (94 tracheal and 12 mainstem), for an overall agreement of 0.83. The sensitivity of ultrasound was 0.91 for tracheal placement of the ETT and 0.50 for mainstem intubation. Notably chest radiography results took a median of 8 min longer to obtain than ultrasound. The study concluded that although diaphragmatic ultrasound was not equivalent to chest radiography for ETT placement within the airway, the results were timelier, detected more misplacements than standard clinical confirmation alone, and were highly reproducible between sonographers.

Lung sliding refers to the dynamic shimmering movement observed at the pleural line, representing apposition and motion of the visceral and parietal pleura during respiration [83]. When a linear or curvilinear transducer is placed on the anterior chest wall, the presence of bilateral lung sliding indicates ventilation of both lungs, supporting correct endotracheal intubation. Absence of bilateral lung sliding raises concern for esophageal intubation, while unilateral absence suggests endobronchial intubation. Although lung sliding has been validated primarily in adult populations, pediatric studies and guidelines support its feasibility in infants and children [75, 83]. In pediatric patients, lung sliding may be more difficult to appreciate due to lower tidal volumes, more rapid respiratory rates, or underlying lung pathology, particularly in neonates. Given the potential limitations of the various US approaches, an integrated US strategy combining transtracheal visualization of the ETT, bilateral lung sliding, and diaphragmatic movement has been proposed to maximize diagnostic accuracy and supplement current clinical methods to validate ETT placement. Ultrasound confirmation of endotracheal intubation may be particularly advantageous in pediatric patients, a population in whom traditional confirmation methods such as auscultation and ETCO_2_ monitoring have notable limitations. Ultrasound should be viewed as a complementary tool that enhances, rather than replaces, established confirmation methods such as waveform capnography. Ultrasound identification of lung sliding may be useful as a primary technique to demonstrate effective lung separation during OLV.

## Identification of the CTM and Cricothyrotomy

Emergency front-of-neck access (eFONA) represents a rare, but potentially life-saving intervention in pediatric airway management, most commonly in the context of a “cannot intubate, cannot oxygenate/ventilate” scenario. Accurate identification of the CTM is crucial to the success of emergency airway access procedures. The pediatric larynx differs significantly from that of adults, the larynx is positioned more cephalad and anterior, the thyroid cartilage is less prominent, the cricoid cartilage represents the narrowest portion of the airway, and the CTM is short in vertical height [84]. These features make accurate identification and palpation of the CTM difficult and contribute to higher rates of landmark misidentification in pediatric patients. Studies have demonstrated that palpation-based identification of the CTM in children is unreliable, even among experienced clinicians [84–86].

Ultrasound identification of the CTM in pediatric patients is performed using a high-frequency linear transducer (10–15 MHz). The probe may be placed either transversely (short-axis) or longitudinally (long-axis) along the anterior neck. In the transverse view, the thyroid cartilage and cricoid cartilage appear as hypoechoic structures with posterior acoustic shadowing, with the CTM visualized as a hyperechoic band between them. In the longitudinal midline view, the thyroid cartilage, CTM, and cricoid cartilage can be visualized sequentially in a single plane, facilitating precise localization [84, 86]. Once identified, the CTM may be marked on the skin to allow rapid access should emergency airway intervention become necessary [84].

**Figure 9:**
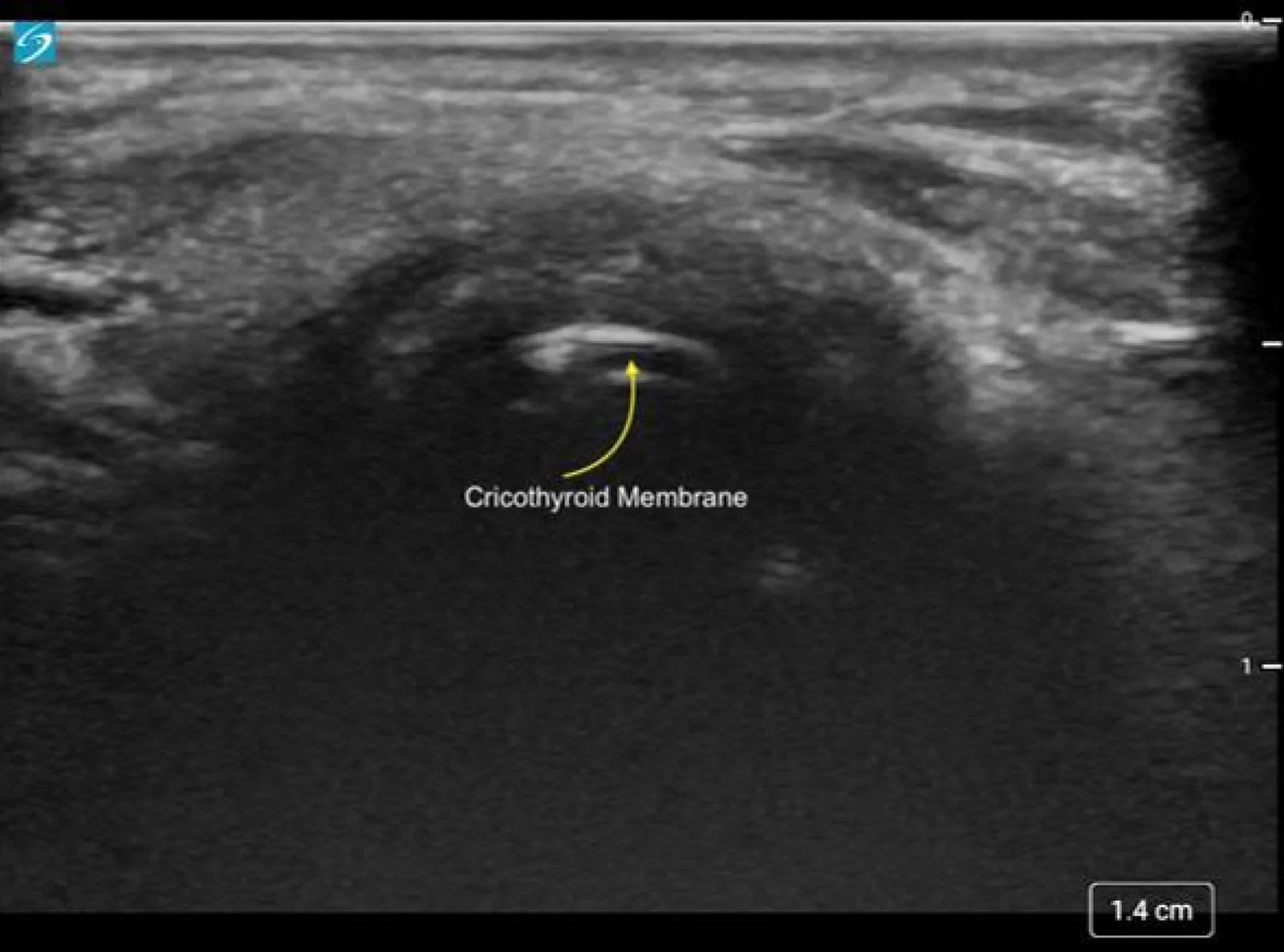
Transverse ultrasound of the neck demonstrating the cricothyroid membrane.

In pediatric anesthesia and critical care medicine, the principal clinical application of ultrasound-guided CTM identification lies in pre-emptive marking of the CTM in children with anticipated difficult airways (Fig. 9). This strategy may reduce delays and uncertainty during airway management with cricothyrotomy, improving readiness, and decreasing procedural time. Ultrasound may also serve as a useful adjunct to airway planning and elective tracheostomy performance, particularly in patients with abnormal anatomy, obesity, or prior neck surgery [86, 87]. Its role as an educational tool has been established, enhancing the clinicians’ understanding of pediatric airway anatomy and reducing reliance on palpation, which is frequently inaccurate in younger children. Despite the advantages of ultrasound-guided CTM identification, several important limitations must be emphasized in pediatric practice. The CTM in infants and small children is extremely narrow, and even with ultrasound, surgical cricothyrotomy is challenging given the small size of the CTM and the pliability of laryngeal structures. As such, it may be difficult to employ successfully in the emergent setting of the “cannot intubate/cannot ventilate” scenario. For a full review of current recommendations for approaches to the “cannot intubate/cannot ventilate” scenario in pediatric patients, the reader is referred to Reference [88].

Surgical cricothyroidotomy may be considered in older children and adolescents, in whom airway dimensions more closely resemble those of adults and the CTM is sufficiently developed. The most appropriate application of airway ultrasound lies in anticipatory use rather than real-time emergency application. Furthermore, accurate identification of the CTM using ultrasound is dependent on operator experience and familiarity with pediatric airway sonography, which limit its effectiveness in untrained hands. Taken together, these considerations underscore that ultrasound-guided identification of the CTM in pediatric patients should be viewed as a pre-emptive and adjunctive strategy, integrated within established pediatric difficult airway algorithms rather than as a replacement for them [88].

## Ultrasound for Assessment of OLV in Pediatric Patients

Accurate confirmation of OLV is critical in pediatric thorax and thoracoscopic surgery as it optimizes surgical exposure, providing a motionless operative lung, while protecting the non-operative lung. Conventional confirmation methods, such as auscultation or fiberoptic bronchoscopy (FOB), may be limited in children due to small airway size, smaller lumens of double lumen ETT, or limited availability of appropriately sized equipment. In this setting, lung ultrasound has emerged as a practical, non-invasive adjunct for real-time assessment of effective lung isolation. Confirmation of lung separation using POCUS can be performed with both the brightness-mode (B-mode) and motion-mode (M-mode). Lung sliding is used in B-mode while the seashore sign and the bar-code sign are important in M-mode [89]. Lung sliding is the ultrasound visualization of the visceral and parietal pleura moving against each other during respiration. The hyperechoic line has been described as a shimmering motion at the pleural line. In the ventilated lung, there is a synchronized movement of these lines with ventilation which corresponds to the movement of the lung or the lung sliding sign. The seashore sign and the bar-code sign can be identified in M-mode. If the lung is ventilated, the seashore sign will be seen while the bar-code sign appears in the absence of ventilation. The M-mode may be more helpful in older patients than in infants and young children because a higher heart rate creates noise in the Bar-code sign which may be misinterpreted as seashore sign.

The published experience in pediatric-aged patients, although initially limited to case reports and small series, supports the applicability of ultrasound in this clinical scenario to identify effective lung isolation with OLV for thoracic procedures. The first six of these published reports were single case reports appearing in the literature from 2015 to 2020 [90–95]. Several of these reports were in neonates or young infants where OLV may be challenging. Ultrasound was suggested to be more sensitive than auscultation for confirming ventilation and lung exclusion. Subsequently, two groups of investigators provided additional information in larger patient cohorts. Tognon et al reported a series of 22 procedures in 20 pediatric patients in which ultrasound was used to confirm effective OLV during video-assisted thoracoscopic surgery [96]. The authors correctly defined lung exclusion by noting the absence of lung sliding with the preservation of lung pulse on the operative side in all cases. Sonographic findings were concordant with direct thoracoscopic visualization. Following OLV, ultrasound confirmed re-expansion and ventilation of the excluded lung, suggesting that US is a safe, rapid, and time-efficient verification method in children. More recently, Moharir et al conducted a prospective blinded observational feasibility study comparing lung US with clinical auscultation for confirmation of OLV in 40 patients ranging in age from 0 to 20 years [97]. After anesthetic induction and placement of the device for OLV, lung separation was confirmed by the primary anesthesia team using standard practice (FOB). A sonographer and auscultator, who were blinded to the FOB findings, then independently assessed lung isolation prior to surgery. Definitive confirmation of lung isolation was obtained by direct intraoperative visualization of lung collapse. Lung ultrasound demonstrated a diagnostic accuracy of 95% (95% CI, 82.7–98.5%), significantly higher than auscultation, which showed an accuracy of 68% (95% CI, 51.5–80.4%) (P < 0.001). Although US required a slightly longer assessment time, it provided substantially greater diagnostic reliability, supporting its role as a superior adjunct to auscultation in pediatric thoracic anesthesia.

Lung ultrasound can be used as an effective adjunct to auscultation to confirm effective lung separation during thoracic surgery. The reader is referred to References [89, 91, 93] for additional images demonstrating the ultrasonographic findings for lung ultrasound during OLV. Keys to obtaining effective ultrasound imaging with additional pearls to guide imaging techniques have been previously outlined by Yamaguchi et al [89]. Imaging begins with obtaining the Bat sign which is used to define the pleural line. Manual ventilation may make it easier to differentiate lung sliding from lung pulse. The cardiac shadow should not be included in the imaging as cardiac motion interferes with focus on the pleural line. Lung ventilation is identified by lung sliding in B-mode and the “seashore sign” in the M-mode. Lung ultrasound is non-invasive, radiation-free, and more accurate than auscultation for confirming lung isolation.

## Summary

Ultrasound has seen a significant expansion of its application in anesthesiology and critical care arenas since its initial introduction to facilitate central venous access. There continues to be an ever-expanding role of POCUS as a practical, rapid, non-invasive, and increasingly indispensable adjunct in airway management in both adult and pediatric patients (Table 2). Beyond its traditional applications in vascular access and regional anesthesia, ultrasound now provides real-time, non-invasive visualization of airway anatomy and dynamic function. The literature demonstrates its utility in preoperative airway assessment, prediction of difficult direct laryngoscopy, confirmation of ETT placement, optimization of ETT cuff position and insertion depth, and selection of appropriate ETT size based on subglottic diameter. Ultrasound also shows promise in identifying airway pathology, assisting in the evaluation of OSA, confirming OLV, and localizing the CTM for anticipated emergency airway access. While pediatric-specific data continue to evolve, current evidence supports ultrasound as a safe, radiation-free, and rapid bedside tool that enhances anatomical understanding and serves as an adjunct to physical assessment. Its role is also expanding in the ICU setting to assess lung and pleural pathology including pneumonia, acute respiratory distress syndrome, pneumothorax, and pleural effusions [98–101]. When integrated with established clinical assessment methods and airway algorithms, POCUS strengthens diagnostic accuracy and may reduce airway-related morbidity in infants and children.

**Table 2:** Key Articles Regarding Airway Ultrasound

First author and reference	Study cohort	Findings
Kundra et al [5]	Review article	Educational review outlining the use of ultrasound (US) in various aspects of airway management including identification of the difficult airway as well as confirmation of endotracheal tube (ETT) placement, prediction of post-extubation stridor, evaluation of soft tissue masses in the neck, and assessment of subglottic diameter for ETT size.
Carsetti et al [15]	Meta-analysis of 15 studies using airway ultrasound to predict difficult intubation.	Airway US index tests are significantly different between patients with easy versus difficult direct laryngoscopy. Distance from the skin to epiglottis (DSE) is the most studied index test in the literature.
Singh et al [35]	Systematic review and meta-analysis of 21 studies in adults (7 airway and 14 non-airway) with 3,339 patients.	Obstructive sleep apnea (moderate-severe) correlated to a moderate degree with the distance between lingual arteries, resting tongue thickness, and tongue base thickness. The non-airway parameter of carotid intimal media thickness had a low to moderate correlation with moderate to severe OSA.
Husein et al [57]	A prospective, double-blinded pediatric study comparing US, video bronchoscopy, and ETT sizing for measurement of subglottic airway diameter.	US measurements correlated with video-bronchoscopy and ETT sizing. However, US underestimated absolute subglottic dimensions. Despite the authors being less enthusiastic about use of US for absolute airway sizing or ETT selection, they opined that it may serve as a useful non-invasive tool for longitudinal monitoring of changes in subglottic caliber over time.
Wani et al [61]	Prospective, non-randomized study of 80 pediatric patients (1–78 months of age).	US used to locate the ETT cuff relative to the cricoid and tracheal rings. The cephalad end of the ETT cuff was found at the level of the cricoid in 16.3% of patients, at the first, second, and third tracheal rings in 27.5%, 23.8%, and 17.5% of patients, respectively, and at or below the 4th tracheal ring in 15% of patients. The authors suggested that this observed inconsistency highlights known variability in pediatric airway anatomy, underscoring the value of real-time US imaging to document cuff position.
Singh et al [68]	Prospective study of 100 pediatric patients (12–60 months of age).	Using US, tracheal diameter was measured as the transverse air column diameter at the cephalad end of the cricoid cartilage. US correlated more strongly with actual ETT size than other formulas (age-based, body length, or finger width).
Marciniak et al [80]	Prospective study of 30 pediatric patients (mean age 48 months).	US imaging was used to visualize passage of the ETT into the airway by visualization and identification of the trachea, tracheal rings, and the vocal cords. The authors noted widening of glottis as the ETT passed through the airway and correctly positioned the ETT by using lung sliding. A single case of esophageal intubation was readily recognized by visualization of the ETT in the esophagus in the left paratracheal space.
Kerrey et al [82]	Lung US versus chest radiography in 127 intubated pediatric patients.	Chest radiography identified the ETT in the mainstem bronchus in 24 patients, while there were no esophageal intubations. US and chest radiography agreed on ETT placement in 106 patients (94 tracheal and 12 mainstem), for an overall agreement of 0.83. Although diaphragmatic US was not equivalent to chest radiography for ETT placement within the airway, the results were timelier, detected more misplacements than standard clinical confirmation alone, and were highly reproducible.
Walsh et al [84]	Prospective study of 22 pediatric patients.	The cricothyroid membrane (CTM) was accurately identified by US in all cases. Height of the CTM correlated with height measured by MRI.
Moharir et al [97]	Prospective, blinded study comparing US with auscultation during one-lung ventilation (OLV) in patients 0–20 years of age.	Lung US demonstrated a diagnostic accuracy of 95%, significantly higher than auscultation (68%, P < 0.001). Although US required a slightly longer assessment time, it provided substantially greater diagnostic reliability, supporting its role as a superior adjunct to auscultation to document effective lung isolation.

## Data Availability

The data supporting the findings of this study are available from the corresponding author upon reasonable request.

## References

[1] Kalagara H, Coker B, Gerstein NS, Kukreja P, Deriy L, Pierce A, Townsley MM (2022). Point-of-Care Ultrasound (POCUS) for the cardiothoracic anesthesiologist. J Cardiothorac Vasc Anesth.

[2] Haskins SC, Vaz AM, Garvin S (2018). Perioperative point-of-care ultrasound for the anesthesiologist. J Anesth Perioper Med.

[3] Tobias JD, Martin DP, Bhalla T (2015). Ultrasound-guided peripheral venous and arterial cannulation in the pediatric population. Anaesth Pain Intensive Care.

[4] Gottlieb M, Holladay D, Burns KM, Nakitende D, Bailitz J (2020). Ultrasound for airway management: An evidence-based review for the emergency clinician. Am J Emerg Med.

[5] Kundra P, Mishra SK, Ramesh A (2011). Ultrasound of the airway. Indian J Anaesth.

[6] Nee PA, Benger J, Walls RM (2008). Airway management. Emerg Med J.

[7] Krishna SG, Tobias JD (2014). An update of airway management in infants and children. Anaesth Pain Intensive Care.

[8] Diaz-Gomez JL, Mayo PH, Koenig SJ (2021). Point-of-care ultrasonography. N Engl J Med.

[9] Gomes SH, Simoes AM, Nunes AM, Pereira MV, Teoh WH, Costa PS, Kristensen MS (2021). Useful ultrasonographic parameters to predict difficult laryngoscopy and difficult tracheal intubation-a systematic review and meta-analysis. Front Med (Lausanne).

[10] Sahu AK, Bhoi S, Aggarwal P, Mathew R, Nayer J, T AV, Mishra PR (2020). Endotracheal tube placement confirmation by ultrasonography: a systematic review and meta-analysis of more than 2500 patients. J Emerg Med.

[11] Chou HC, Tseng WP, Wang CH, Ma MH, Wang HP, Huang PC, Sim SS (2011). Tracheal rapid ultrasound exam (T.R.U.E.) for confirming endotracheal tube placement during emergency intubation. Resuscitation.

[12] Ramsingh D, Frank E, Haughton R, Schilling J, Gimenez KM, Banh E, Rinehart J (2016). Auscultation versus Point-of-care Ultrasound to Determine Endotracheal versus Bronchial Intubation: A Diagnostic Accuracy Study. Anesthesiology.

[13] Alerhand S (2018). Ultrasound for identifying the cricothyroid membrane prior to the anticipated difficult airway. Am J Emerg Med.

[14] Lin J, Bellinger R, Shedd A, Wolfshohl J, Walker J, Healy J, Taylor J (2023). Point-of-care ultrasound in airway evaluation and management: a comprehensive review. Diagnostics (Basel).

[15] Carsetti A, Sorbello M, Adrario E, Donati A, Falcetta S (2022). Airway ultrasound as predictor of difficult direct laryngoscopy: a systematic review and meta-analysis. Anesth Analg.

[16] Peterson GN, Domino KB, Caplan RA, Posner KL, Lee LA, Cheney FW (2005). Management of the difficult airway: a closed claims analysis. Anesthesiology.

[17] Cook TM, Woodall N, Frerk C, Fourth National Audit P (2011). Major complications of airway management in the UK: results of the Fourth National Audit Project of the Royal College of Anaesthetists and the Difficult Airway Society. Part 1: anaesthesia. Br J Anaesth.

[18] Pandit JJ, Heidegger T (2017). Putting the 'point' back into the ritual: a binary approach to difficult airway prediction. Anaesthesia.

[19] Shiga T, Wajima Z, Inoue T, Sakamoto A (2005). Predicting difficult intubation in apparently normal patients: a meta-analysis of bedside screening test performance. Anesthesiology.

[20] Roth D, Pace NL, Lee A, Hovhannisyan K, Warenits AM, Arrich J, Herkner H (2018). Airway physical examination tests for detection of difficult airway management in apparently normal adult patients. Cochrane Database Syst Rev.

[21] L'Hermite J, Nouvellon E, Cuvillon P, Fabbro-Peray P, Langeron O, Ripart J (2009). The Simplified Predictive Intubation Difficulty Score: a new weighted score for difficult airway assessment. Eur J Anaesthesiol.

[22] Seo SH, Lee JG, Yu SB, Kim DS, Ryu SJ, Kim KH (2012). Predictors of difficult intubation defined by the intubation difficulty scale (IDS): predictive value of 7 airway assessment factors. Korean J Anesthesiol.

[23] Ohri R, Malhotra K (2020). Different 2D ultrasound calculation methods to evaluate tongue volume for prediction of difficult laryngoscopy. Indian J Anaesth.

[24] Riveros-Perez E, Avella-Molano B, Rocuts A (2025). Airway ultrasound: a narrative review of present use and future applications in anesthesia. Healthcare (Basel).

[25] Bhargava V, Rockwell NA, Tawfik D, Haileselassie B, Petrisor C, Su E (2023). Prediction of difficult laryngoscopy using ultrasound: a systematic review and meta-analysis. Crit Care Med.

[26] Kanoujiya J, Sancheti A, Swami S (2019). Prediction of difficult laryngoscopy by ultrasound-guided evaluation of anterior neck soft tissue thickness. Int J Adv Res.

[27] Adhikari S, Zeger W, Schmier C, Crum T, Craven A, Frrokaj I, Pang H (2011). Pilot study to determine the utility of point-of-care ultrasound in the assessment of difficult laryngoscopy. Acad Emerg Med.

[28] Fernandez-Vaquero MA, Charco-Mora P, Garcia-Aroca MA, Greif R (2023). Preoperative airway ultrasound assessment in the sniffing position: a prospective observational study. Braz J Anesthesiol.

[29] Ni H, Guan C, He G, Bao Y, Shi D, Zhu Y (2020). Ultrasound measurement of laryngeal structures in the parasagittal plane for the prediction of difficult laryngoscopies in Chinese adults. BMC Anesthesiol.

[30] Kahlon S, Jain D, Bhardwaj N, Gandhi K, Jafra A (2023). Ultrasound evaluation to predict difficult laryngoscopy in children below 2 years. Indian J Pediatr.

[31] Zheng Z, Wang X, Du R, Wu Q, Chen L, Ma W (2023). Effectiveness of ultrasonic measurement for the hyomental distance and distance from skin to epiglottis in predicting difficult laryngoscopy in children. Eur Radiol.

[32] Stafrace S, Engelhardt T, Teoh WH, Kristensen MS (2016). Essential ultrasound techniques of the pediatric airway. Paediatr Anaesth.

[33] Osman A, Sum KM (2016). Role of upper airway ultrasound in airway management. J Intensive Care.

[34] Shaikh F, Al-Amir A (2020). Upper airway ultrasound: a promising tool in the management of pediatric airways. J Pediatr Crit Care.

[35] Singh M, Tuteja A, Wong DT, Goel A, Trivedi A, Tomlinson G, Chan V (2019). Point-of-care ultrasound for obstructive sleep apnea screening: Are we there yet? A systematic review and meta-analysis. Anesth Analg.

[36] Burns DW, Chan VWS, Trivedi A, Englesakis M, Munshey F, Singh M (2022). Ready to scan? A systematic review of point of care ultrasound (PoCUS) for screening of obstructive sleep apnea (OSA) in the pediatric population. J Clin Anesth.

[37] Li HY, Lee LA (2009). Sleep-disordered breathing in children. Chang Gung Med J.

[38] Berry RB, Budhiraja R, Gottlieb DJ, Gozal D, Iber C, Kapur VK, Marcus CL (2012). Rules for scoring respiratory events in sleep: update of the 2007 AASM Manual for the Scoring of Sleep and Associated Events. Deliberations of the Sleep Apnea Definitions Task Force of the American Academy of Sleep Medicine. J Clin Sleep Med.

[39] Hwang M, Zhang K, Nagappa M, Saripella A, Englesakis M, Chung F (2021). Validation of the STOP-Bang questionnaire as a screening tool for obstructive sleep apnoea in patients with cardiovascular risk factors: a systematic review and meta-analysis. BMJ Open Respir Res.

[40] Simpson L, Hillman DR, Cooper MN, Ward KL, Hunter M, Cullen S, James A (2013). High prevalence of undiagnosed obstructive sleep apnoea in the general population and methods for screening for representative controls. Sleep Breath.

[41] Lin CY, Chen CN, Kang KT, Hsiao TY, Lee PL, Hsu WC (2018). Ultrasonographic evaluation of upper airway structures in children with obstructive sleep apnea. JAMA Otolaryngol Head Neck Surg.

[42] Au CT, Chan KCC, Liu KH, Chu WCW, Wing YK, Li AM (2018). Potential anatomic markers of obstructive sleep apnea in prepubertal children. J Clin Sleep Med.

[43] Au CT, Chan KC, Zhang J, Liu KH, Chu WCW, Wing YK, Li AM (2021). Intermediate phenotypes of childhood obstructive sleep apnea. J Sleep Res.

[44] Mitchell RB, Archer SM, Ishman SL, Rosenfeld RM, Coles S, Finestone SA, Friedman NR (2019). Clinical practice guideline: tonsillectomy in children (update)-executive summary. Otolaryngol Head Neck Surg.

[45] Katz ES, D'Ambrosio CM (2008). Pathophysiology of pediatric obstructive sleep apnea. Proc Am Thorac Soc.

[46] Zhou M, Guo B, Wang Y, Yan D, Lin C, Shi Z (2017). The association between obstructive sleep apnea and carotid intima-media thickness: a systematic review and meta-analysis. Angiology.

[47] Song F, Zou J, Song Z, Xu H, Qian Y, Zhu H, Liu S (2020). Association of adipocytokines with carotid intima media thickness and arterial stiffness in obstructive sleep apnea patients. Front Endocrinol (Lausanne).

[48] Drager LF, Bortolotto LA, Lorenzi MC, Figueiredo AC, Krieger EM, Lorenzi-Filho G (2005). Early signs of atherosclerosis in obstructive sleep apnea. Am J Respir Crit Care Med.

[49] Iannuzzi A, Licenziati MR, De Michele F, Verga MC, Santoriello C, Di Buono L, Renis M (2013). C-reactive protein and carotid intima-media thickness in children with sleep disordered breathing. J Clin Sleep Med.

[50] Marshall NS, Ayer JG, Toelle BG, Harmer JA, Phillips CL, Grunstein RR, Celermajer DS (2011). Snoring is not associated with adverse effects on blood pressure, arterial structure or function in 8-year-old children: the Childhood Asthma Prevention Study (CAPS). J Paediatr Child Health.

[51] Tagetti A, Bonafini S, Zaffanello M, Benetti MV, Vedove FD, Gasperi E, Cavarzere P (2017). Sleep-disordered breathing is associated with blood pressure and carotid arterial stiffness in obese children. J Hypertens.

[52] Giguere CM, Manoukian JJ, Patenaude Y, Platt R (2000). Ultrasound and a new videobronchoscopic technique to measure the subglottic diameter. J Otolaryngol.

[53] Miles KA (1989). Ultrasound demonstration of vocal cord movements. Br J Radiol.

[54] Kundra P, Kumar K, Allampalli V, Anathkrishnan R, Gopalakrishnan S, Elangovan S (2012). Use of ultrasound to assess superior and recurrent laryngeal nerve function immediately after thyroid surgery. Anaesthesia.

[55] Garel C, Hassan M, Legrand I, Elmaleh M, Narcy P (1991). Laryngeal ultrasonography in infants and children: pathological findings. Pediatr Radiol.

[56] Bisetti MS, Segala F, Zappia F, Albera R, Ottaviani F, Schindler A (2009). Non-invasive assessment of benign vocal folds lesions in children by means of ultrasonography. Int J Pediatr Otorhinolaryngol.

[57] Husein M, Manoukian JJ, Platt R, Patenaude Y, Drouin S, Giguere C (2002). Ultrasonography and videobronchoscopy to assess the subglottic diameter in the paediatric population: a first look. J Otolaryngol.

[58] Bell JR, Cohen AP, Graff JT, Fleck RJ, O'Hara S, de Alarcon A, Hart CK (2020). Pilot study to assess the use of ultrasound in evaluating the abnormal pediatric airway. Otolaryngol Head Neck Surg.

[59] Or DY, Karmakar MK, Lam GC, Hui JW, Li JW, Chen PP (2013). Multiplanar 3D ultrasound imaging to assess the anatomy of the upper airway and measure the subglottic and tracheal diameters in adults. Br J Radiol.

[60] Bhardwaj N (2013). Pediatric cuffed endotracheal tubes. J Anaesthesiol Clin Pharmacol.

[61] Wani TM, John J, Rehman S, Bhaskar P, Sahabudheen AF, Mahfoud ZR, Tobias JD (2021). Point-of-care ultrasound to confirm endotracheal tube cuff position in relationship to the cricoid in the pediatric population. Paediatr Anaesth.

[62] Isa M, Holzki J, Hagemeier A, Rothschild MA, Cote CJ (2021). Anatomical in vitro investigations of the pediatric larynx: a call for manufacturer redesign of tracheal tube cuff location and perhaps a call to reconsider the use of uncuffed tracheal tubes. Anesth Analg.

[63] Levkovitz O, Schujovitzky D, Stackievicz R, Fayoux P, Morag I, Litmanovitz I, Arnon S (2023). Ultrasound assessment of endotracheal tube depth in neonates: a prospective feasibility study. Arch Dis Child Fetal Neonatal Ed.

[64] Salvadori S, Nardo D, Frigo AC, Oss M, Mercante I, Moschino L, Priante E (2021). Ultrasound for endotracheal tube tip position in term and preterm infants. Neonatology.

[65] Congedi S, Savio F, Auciello M, Salvadori S, Nardo D, Bonadies L (2022). Sonographic evaluation of the endotracheal tube position in the neonatal population: a comprehensive review and meta-analysis. Front Pediatr.

[66] Uya A, Gautam NK, Rafique MB, Pawelek O, Patnana SR, Gupta-Malhotra M, Balaguru D (2020). Point-of-care ultrasound in sternal notch confirms depth of endotracheal tube in children. Pediatr Crit Care Med.

[67] Liu Y, Ma W, Liu J (2023). Applications of airway ultrasound for endotracheal intubation in pediatric patients: a systematic review. J Clin Med.

[68] Singh S, Jindal P, Ramakrishnan P, Raghuvanshi S (2019). Prediction of endotracheal tube size in children by predicting subglottic diameter using ultrasonographic measurement versus traditional formulas. Saudi J Anaesth.

[69] Hao J, Zhang J, Dong B, Luo Z (2020). The accuracy of ultrasound to predict endotracheal tube size for pediatric patients with congenital scoliosis. BMC Anesthesiol.

[70] Kim EJ, Kim SY, Kim WO, Kim H, Kil HK (2013). Ultrasound measurement of subglottic diameter and an empirical formula for proper endotracheal tube fitting in children. Acta Anaesthesiol Scand.

[71] Shibasaki M, Nakajima Y, Ishii S, Shimizu F, Shime N, Sessler DI (2010). Prediction of pediatric endotracheal tube size by ultrasonography. Anesthesiology.

[72] Ahn JH, Park JH, Kim MS, Kang HC, Kim IS (2021). Point of care airway ultrasound to select tracheal tube and determine insertion depth in cleft repair surgery. Sci Rep.

[73] Gottlieb M, Alerhand S, Long B (2020). Point-of-care ultrasound for intubation confirmation of COVID-19 patients. West J Emerg Med.

[74] Zechner PM, Breitkreutz R (2011). Ultrasound instead of capnometry for confirming tracheal tube placement in an emergency?. Resuscitation.

[75] Singh Y, Tissot C, Fraga MV, Yousef N, Cortes RG, Lopez J, Sanchez-de-Toledo J (2020). International evidence-based guidelines on Point of Care Ultrasound (POCUS) for critically ill neonates and children issued by the POCUS Working Group of the European Society of Paediatric and Neonatal Intensive Care (ESPNIC). Crit Care.

[76] Gottlieb M, Holladay D, Peksa GD (2018). Ultrasonography for the confirmation of endotracheal tube intubation: a systematic review and meta-analysis. Ann Emerg Med.

[77] Sustic A (2007). Role of ultrasound in the airway management of critically ill patients. Crit Care Med.

[78] Hoffmann B, Gullett JP, Hill HF, Fuller D, Westergaard MC, Hosek WT, Smith JA (2014). Bedside ultrasound of the neck confirms endotracheal tube position in emergency intubations. Ultraschall Med.

[79] Muslu B, Sert H, Kaya A, Demircioglu RI, Gozdemir M, Usta B, Boynukalin KS (2011). Use of sonography for rapid identification of esophageal and tracheal intubations in adult patients. J Ultrasound Med.

[80] Marciniak B, Fayoux P, Hebrard A, Krivosic-Horber R, Engelhardt T, Bissonnette B (2009). Airway management in children: ultrasonography assessment of tracheal intubation in real time?. Anesth Analg.

[81] Chen WT, Wang MY, Jiang TT, Tang M, Ye QH, Wang HY, Mo EJ (2022). Transtracheal ultrasound for confirmation of endotracheal tube placement in the intensive care unit: a systematic review and meta-analysis. Eur Rev Med Pharmacol Sci.

[82] Kerrey BT, Geis GL, Quinn AM, Hornung RW, Ruddy RM (2009). A prospective comparison of diaphragmatic ultrasound and chest radiography to determine endotracheal tube position in a pediatric emergency department. Pediatrics.

[83] Lichtenstein DA, Menu Y (1995). A bedside ultrasound sign ruling out pneumothorax in the critically ill. Lung sliding. Chest.

[84] Walsh B, Fennessy P, Ni Mhuircheartaigh R, Snow A, McCarthy KF, McCaul CL (2019). Accuracy of ultrasound in measurement of the pediatric cricothyroid membrane. Paediatr Anaesth.

[85] Fennessy P, Walsh B, Laffey JG, McCarthy KF, McCaul CL (2020). Accuracy of pediatric cricothyroid membrane identification by digital palpation and implications for emergency front of neck access. Paediatr Anaesth.

[86] Cho SA, Kang P, Song IS, Ji SH, Jang YE, Lee JH, Kim JT (2022). Performance time of anesthesiology trainees for cricothyroid membrane identification and characteristics of cricothyroid membrane in pediatric patients using ultrasonography. Paediatr Anaesth.

[87] Berger-Estilita J, Wenzel V, Luedi MM, Riva T (2021). A primer for pediatric emergency front-of-the-neck access. A A Pract.

[88] Black AE, Flynn PE, Smith HL, Thomas ML, Wilkinson KA, Association of Pediatric Anaesthetists of Great, Britain Ireland (2015). Development of a guideline for the management of the unanticipated difficult airway in pediatric practice. Paediatr Anaesth.

[89] Yamaguchi Y, Moharir A, Burrier C, Nomura T, Tobias JD (2020). Point-of-care lung ultrasound to evaluate lung isolation during one-lung ventilation in children: narrative review. Med Devices (Auckl).

[90] Nam JS, Park I, Seo H, Min HG (2015). The use of lung ultrasonography to confirm lung isolation in an infant who underwent emergent video-assisted thoracoscopic surgery: a case report. Korean J Anesthesiol.

[91] Yamaguchi Y, Moharir A, Burrier C, Tobias JD (2019). Point-of-care lung ultrasound to evaluate lung isolation during one-lung ventilation in children: a case report. Saudi J Anaesth.

[92] Adler AC (2019). Extraluminal use of a pediatric bronchial blocker with confirmation by point-of-care ultrasound: a case report. A A Pract.

[93] Yamaguchi Y, Moharir A, Nomura T, Tobias JD (2019). Point-of-care lung ultrasound for bronchial blocker placement. A A Pract.

[94] Rodrigues A, Alves P, Hipolito C, Salgado H (2019). [Will ultrasound replace the stethoscope?: a case report on neonatal one-lung ventilation]. Braz J Anesthesiol.

[95] De Marchi L, Patel J, Razmjou K (2020). Lung ultrasound in thoracic surgery: confirming placement of a pediatric right double-lumen tube. A A Pract.

[96] Tognon C, Pulvirenti R, Pizzi S, Zuliani M, Cortese G, Esposito C, Gamba P (2022). Lung ultrasound to assess one lung ventilation: a pediatric case series. J Laparoendosc Adv Surg Tech A.

[97] Moharir A, Yamaguchi Y, Aldrink JH, Martinez A, Arce-Villalobos M, Yemele Kitio SA, Rice-Weimer J (2024). Point-of-care lung ultrasound to evaluate lung isolation during one-lung ventilation in children: a blinded observational feasibility study. Anesth Analg.

[98] Rizvi MB, Rabiner JE (2022). Pediatric point-of-care lung ultrasonography: a narrative review. West J Emerg Med.

[99] Penk JS, Bhargava V, Chandnani H, Chong G, Conlon T, DeSanti RL, Diddle JW (2026). Good practice concepts in pediatric critical care point-of-care ultrasound: a modified delphi consensus initiative. Pediatr Crit Care Med.

[100] DeSanti RL, Al-Subu AM, Cowan EA, Kamps NN, Lasarev MR, Schmidt J, Kory PD (2021). Point-of-care lung ultrasound to diagnose the etiology of acute respiratory failure at admission to the PICU. Pediatr Crit Care Med.

[101] Watkins LA, Dial SP, Koenig SJ, Kurepa DN, Mayo PH (2022). The utility of point-of-care ultrasound in the pediatric intensive care unit. J Intensive Care Med.

